# Epidemiology of visceral leishmaniasis in Shebelle Zone of Somali Region, eastern Ethiopia

**DOI:** 10.1186/s13071-019-3452-5

**Published:** 2019-05-06

**Authors:** Getachew Alebie, Amha Worku, Siele Yohannes, Befikadu Urga, Asrat Hailu, Dagimawie Tadesse

**Affiliations:** 1 Department of Biology, Jigjiga University, Jigjiga, Ethiopia; 2 College of Veterinary Medicine, Jigjiga University, Jigjiga, Ethiopia; 3 Department of Microbiology, Immunology and Parasitology, Faculty of Medicine, Addis Ababa University, Addis Ababa, Ethiopia; 4 Department of Medical Microbiology, DNDi Leishmaniasis Research and Treatment Center, Arbaminch Hospital, Arbaminch, Ethiopia

**Keywords:** Epidemiology, Visceral leishmaniasis, Shebelle Zone, Somali Region

## Abstract

**Background:**

Visceral leishmaniasis (VL), a vector-borne disease caused by species of the *L.donovani* complex, has (re)-emerged in Ethiopia during the last two decades and is currently of increasing public health concern. However, very little is known about VL epidemiology in the Somali Region of Ethiopia. The aim of this study was to provide detailed epidemiological information on seroprevalence, associated factors and incriminated vectors of VL in Shebelle Zone and Ethiopian Somali Region in general.

**Methods:**

A cross-sectional epidemiological study was conducted between March and May 2016 in Gode and Adadle districts of Shebelle Zone, Ethiopian Somali Region. Two-stage semi-random sampling was applied for selecting study participants for the field survey. The study included structured questionnaire interviews, serological assays (rK39-immunochromatographic test), ELISA and entomological surveys.

**Results:**

From a total of 361 participants, 57 (15.8%) were seropositive for VL including 46 (12.7%) rK39 positive and 11 (3.0%) positive by both rK39 and ELISA. VL seroprevalence was higher (*P* < 0.001) in Adadle (31.1%) compared to Gode (12.7%) district. The VL seroprevalence rate was higher in females than in males [rK39 (17.2 *vs* 14.0%) and ELISA (3.4 *vs* 2.5%)]. Children under the 15 years of age were the most highly affected group [rK39 (20.4%) and ELISA (4.4%)]. Increased VL risk was associated with presence of termite hills, study district, outdoor sleeping, *Acacia* trees and domestic animals [odds ratio (95% confidence interval): 12.58 (5.911–26.763), 5.40 (2.90–10.07), 5.31 (2.283–12.364), 2.37 (1.1190–4.728) and 0.199 (0.097–0.410), respectively]. The entomological survey identified 74 *Phlebotomus* [*P.* (*Larroussius*) *orientalis* (52/74), *P.* (*Anaphlebotomus*) *rodhaini* (14/74), *P.* (*Paraphlebotomus*) *sergenti* (8/74)] and 11 *Sergentomyia* sand flies. The average frequency of *P. orientalis* (3.06 ± 0.66) collected by all traps per night was higher than that of other species. The average frequency of total and specific (*P. orientalis*) female sand flies was higher in Adadle (1.89 ± 0.423 *vs* 1.11 ± 0.309) than in Gode (0.62 ± 0.324 *vs* 0.38 ± 0.183) district. The highest mean numbers of total (8 ± 1.5) and *P. orientalis* (6 ± 0.913) sand flies were collected in termite hills.

**Conclusions:**

The present findings revealed potential new VL-transmission foci in the study districts. Therefore, the need for parasitological and molecular characterization of the parasite in humans and vector sand flies is of paramount importance to confirm transmission.

**Electronic supplementary material:**

The online version of this article (10.1186/s13071-019-3452-5) contains supplementary material, which is available to authorized users.

## Background

Visceral leishmaniasis (VL), considered among the most neglected tropical diseases, is one of several emerging diseases of major public health importance in Ethiopia [1]. An estimated 3.2 million people are at risk of VL in Ethiopia and an estimated 375,633 km^2^ (33%) of the landmass in northeastern, northwestern, western and southeastern parts of the country is highly suitable for the transmission of VL [2]. Moreover, recurrent epidemics paralleled by elevated rates of co-infection with HIV (up to about 30%), increased mortality and morbidity rates have occurred in several VL endemic localities of the country [3–7].

Recently, the epidemiology of VL has been changed in the country, with endemic areas continually spreading [6, 8, 9]. This is in-line with the contemporary trend of climate change, rapid urbanization and massive population movements that have altered the range and population density of the insect vectors and reservoir hosts of the disease, resulting in the cumulative elevation in the rate of human exposure to the infection [6, 10–13].

In the Ethiopian Somali Region, VL outbreaks were first reported in 2001 from Afder, Liben Denan and Hagele areas, bordering Kenya and Somalia [8]. Since then, VL cases have been reported sporadically from different localities of the region [2, 8, 14–16]. In March 2010, some blood samples examined by DAT (direct agglutination test) from Shebelle Zone (including Gode and Adadle districts) by MSF‐H (Dutch Section) tested positive for VL [14]. According to the national risk map survey of VL, large areas in the region were predicted to be at high and very high risk of VL, some of which include Barey, Chereti, Danan, Hargele, Dolo-Addo, Dolo Bay, Debe-Woin, East Eme, Elkere, Filtu, Ferfer, Godey, Kelafo, Sagag, Mustahil, Salahad and West Eme districts [2]. Based on the data obtained from Gode general hospital, nine individuals were diagnosed with active VL cases only in the year before this study was conducted, of which seven individuals detected with active VL cases were permanent residents of the two study areas (three cases in Gode and four cases in Adadle).

In spite of such records and associated co-morbidities such as malnutrition and the HIV pandemic, owing to lack of comprehensive data on VL transmission and magnitude therein, the region (including Shebelle Zone) is epidemiologically under-represented at the national level. Therefore, the purpose of the present study was to assess seroepidemiology of VL in Gode and Adadle districts, Shebelle Zone, eastern Ethiopia.

## Methods

### Study areas

**Fig. 1:**
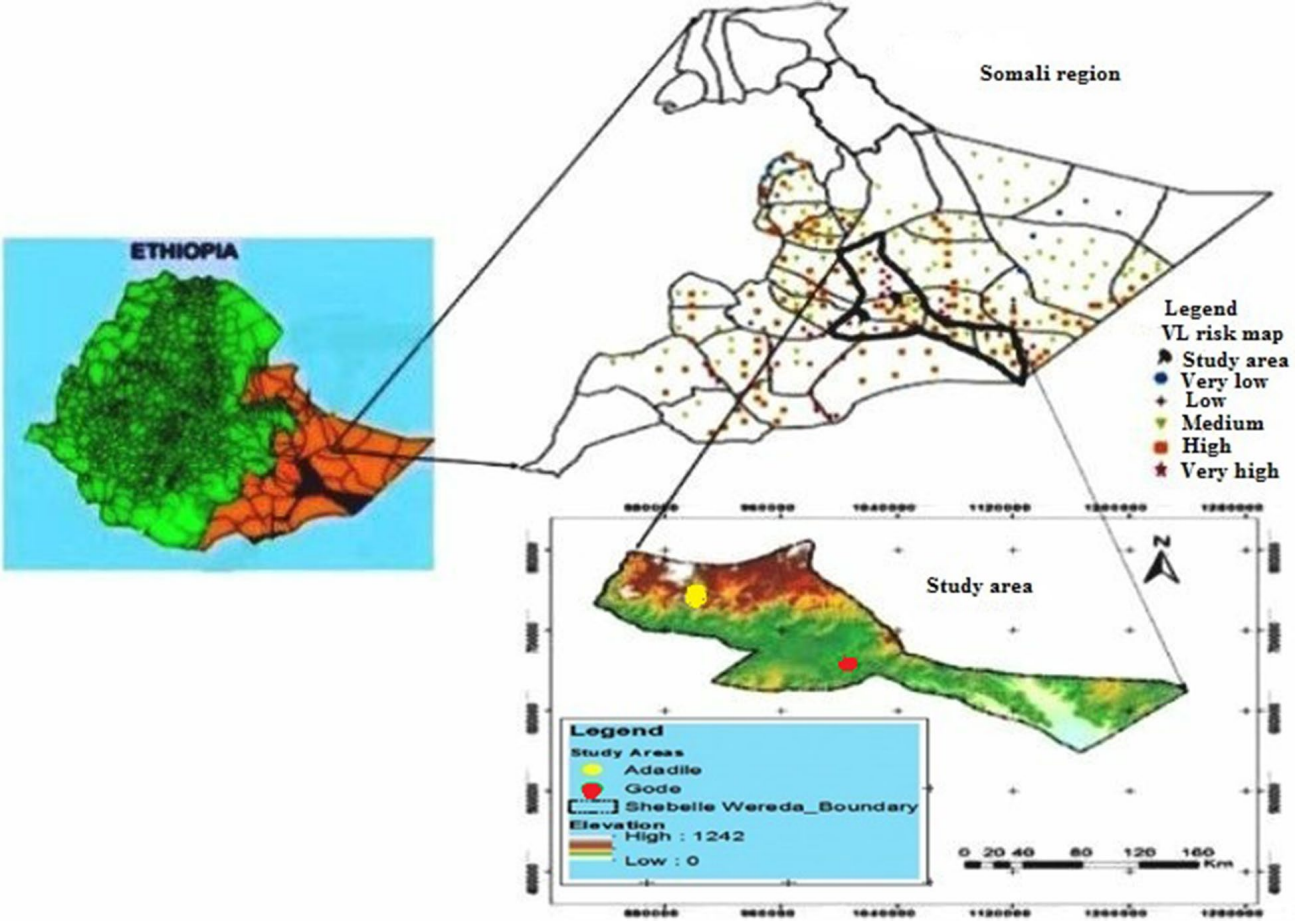
Map showing the study areas. Adapted from I-scholar website, https://www.i-scholar.in/index.php/IJIRI (under Creative Commons license)

This study was conducted in two districts (i.e. Gode and Adadle) of Shebelle Zone, Ethiopian Somali Regional State (Fig. 1). Shebelle Zone, one of the nine zones in the region, is 600 and 1228 km from Jigjiga town and Addis Ababa city, respectively. It is bordered to the southwest by Afder Zone, to the south by Oromia Region, to the north by Fiq Zone and to the northeast by Korahe Zone. It is located at an average altitude of around 300 m above sea level. Shebelle Zone is climatically characterized as arid to semi-arid agro-ecology. The rainfall pattern is characterized by two rainy seasons. The main rainy season extends from April to June (Gu) and the short rainy season from October to December (Deyr). There are also two dry seasons, the long dry season (Hagaa) extends from early July through the end of September and the short dry season (Jilaal) from December through mid-March. The average annual rainfall is around 250–300 mm and the maximum and minimum temperatures are 40 °C and 28 °C, respectively [17, 18]. Gode is the only town in Shebelle Zone and the second largest city next to Jigjiga in Somali Region. The most common livelihoods in the town include employment as day laborers, in trade, remittances, livestock and small-scale agriculture in the nearby rural kebeles. In contrast, Adadle district is in the remote rural center of the district where the vast majorities of villagers maintain livestock for their livelihoods. Being a rural district, a large portion of the range land is covered by vegetation, mainly *Acacia* trees, and numerous termite hills/mounds.

### Study design

Cross-sectional serological and entomological surveys were conducted from March to May 2016 to assess the seroprevalence, potential phlebotomine vectors and associated factors of VL in the study districts. Permanent residents of Gode and Adadle districts were enrolled in the study. Individuals who previously had VL disease were excluded. Two-stage semi-random sampling was applied for selecting study participants for the field survey. In the first stage, study communities were selected on the basis of the data record of VL patients previously clinically admitted in Gode Hospital, the security condition and suitability of the area for transportation. In the second stage, every 5th household was systematically selected based on information from Gode hospital and pastoral settlement patterns and then participants were randomly selected and were requested to come in to Gode Hospital and Adadle Health Center by the next day for blood sample collection.

### Sample size determination

Considering the lack of credible estimates on the magnitude of VL in the region, sample size was estimated using the formula for simple random sampling [19] at 95% confidence interval (z = 1.96), estimated prevalence (p) of 50% and 5% precision level (d):

$$n = \frac{{z^{2} p(1 - p)}}{{d^{2} }}$$

The sample size obtained using the above formula was 384. However, 11 participants were excluded from the study as they refused to give blood sample, whereas blood samples of 12 individuals were discarded due to contamination. Hence, the actual sample size used in this study was 361.

### Blood sample collection

Prior to blood collection, socio-demographic information was gathered from the study participants using a pre-tested structured questionnaire. Thereafter, blood samples (5 ml) were collected from each study participant, and all samples were transported in a cold box to the Medical Parasitology Unit, Department of Microbiology and Immunology of Addis Ababa University for rapid test examination and to the Leishmaniasis Research Station in Konso District, southern Ethiopia for serological (ELISA) examination.

### Sand fly collection

Sand flies were collected, primarily using CDC miniature light traps and sticky traps (ST) consisting of A4-sized polypropylene sheets coated with sesame oil. Sand fly collection began in March and continued through May 2016 to coincide with their second annual population peak [20]. Four light traps were operated from 17:00 h through 6:00 h for two consecutive nights on selected sampling sites (termite hills, trees and cattle sheds) and sticky traps were operated in these outdoor sites and inside the huts of study participants. The distance between the outdoor sites ranged between 150–200 m. In the early morning of each sampling days, sand flies captured inside the mesh-collection bag of each trap were collected with an aspirator. Alongside the above methods, insects that fell down in the artificial ponds of some villagers of Adadle district were collected using an aspirator. Finally, collected specimens were preserved on the same day of collection in 80% ethanol and transported to the laboratory of Jigjiga University’s Biology Department for further examination.

### rK39-immunochromatographic test (rK39-ICT)

Whole blood samples were tested at the medical school of Addis Ababa University using rK39 ICT (DiaMed-ITLEISH; Bio-Rad Laboratories, Marnes-la-Coquette, France) following the recommended procedure supplied with the kit. A 0.5-μl blood sample was added to the absorbent pad well with a drop of buffer provided with the kit. Finally, the results were read after 10–20 min and recorded as follows: positive when both control and test lines appear; negative when only control line appears; invalid when no control line appears.

### ELISA test

The presence of antibodies against *L. infantum/donovani* also was determined by a commercially available *Leishmania* IgM + IgG ELISA (Vircell SL, Granada, Spain) according to the manufacturer’s instructions. All steps were performed at 20–25 °C and under continuous gentle agitation. Briefly, 100 µl of diluted samples (1:25) and controls (negative, high positive and low positive) were added to the appropriate well and incubated for 10 min. Wells were washed five times with 0.3 ml of washing solution and 100 µl of conjugate was added to each well. After 5 min, the wells were washed again and 100 µl of substrate was added, and then incubated for 20 min. The reaction was stopped after 10 min by adding 50 µl of stopping solution. Microplates were read by a microplate spectrophotometer at 450 nm within 1 h of stopping. Results were expressed by comparing the optical density (O.D.) of the sample with the O.D. of the cut-off serum mean included in the kit.

### Sand fly dissections and morphological identification

Sand fly specimens were dissected and mounted on microscope slides in Hoyer’s medium with their heads separate from thoraxes and abdomens. Slide-mounted flies were then identified to species level based on the external genitalia of males and the pharynx, antennal features and spermathecae of females, according to standard morphological keys [21–25].

### Data analysis

All data were entered into the Statistical Software Packages for Social Science software (SPSS) v.22.0. Descriptive statistics was computed to determine frequency and percentage. The Chi-square statistic was used to determine the associations between socio-demographic characteristics and VL positivity. Kappa (κ)-statistics were computed to measure the agreement between the two tests. Independent and paired t-tests were applied to determine the mean differences in frequency of insects in terms of different factors. Logistic regression was used to examine possible factors associated with VL seroprevalence. An adjusted odds ratio was obtained using a CI of 95%. For all included studies, a *P*-value < 0.05 was regarded as statistically significant.

## Results

### Socio-demographic characteristics

A total of 361 participants including 300 from Gode and 61 from Adadle districts were recruited. Overall, a higher proportion of the participants were female (56.5%), < 30 years-old (67.1%) and engaged in lower school study and cattle keeping (39.1%) or agricultural (32.7%) occupation. Likewise, higher proportions of participants belonged to households living in mud and stone-walled dwellings (71.7%) and raised livestock (77.3%) inhabiting localities with abundant termite mounds (54.8%) and *Acacia* tree cover (64.8%). Raising livestock and the presence of termite mounds and *Acacia* trees were more significant factors in Adadle than in Gode district (*P* < 0.050). Meanwhile, dwellings of mud/stone and wooden construction were more frequent (*P* < 0.050) in Gode and Adadle districts, respectively (Table 1).

**Table 1:** Socio-demographic characteristics of participants based on the study districts

Variable	Category	*n* (%)			*χ* ^2^	*P*-value
		Gode	Adadle	Total		
Sex	Female	168 (56.0)	36 (59.0)	204 (56.5)	0.188	0.665
	Male	132 (44.0)	25 (41.0)	157 (43.5)		
Age group	0–14	111 (37.0)	26 (42.6)	137 (38.0)	3.249	0.355
	15–29	89 (29.7)	16 (26.2)	105 (29.1)		
	30–44	67 (22.3)	9 (14.8)	76 (21.1)		
	> 44	33 (11.0)	10 (16.4)	43 (11.9)		
Occupation	Farmer	98 (32.7)	20 (32.8)	118 (32.7)	0.594	0.964
	House worker/wife	53 (17.7)	9 (14.8)	62 (17.2)		
	Student/cattle keeper	115 (38.3)	26 (42.6)	141 (39.1)		
	Employed	11 (3.7)	2 (3.3)	13 (3.6)		
	Private worker	23 (7.7)	4 (6.6)	27 (7.5)		
House type (wall)	Mud/wood	216 (72.0)	43 (70.5)	259 (71.7)	8.428	0.015
	Mud/stone	58 (19.3)	6 (9.8)	64 (17.7)		
	Wood	26 (8.7)	12 (19.7)	38 (10.5)		
Habit of sleeping outdoors	Yes	159 (53.0)	33 (54.1)	192 (53.2)	0.025	0.875
	No	141 (47.0)	28 (45.9)	169 (46.8)		
Presence of domestic animals	Yes	222 (74.0)	57 (93.4)	279 (77.3)	10.916	0.001
	No	78 (26.0)	4 (6.6)	82 (22.7)		
Presence of *Acacia* tree	Yes	125 (41.7)	38 (62.3)	163 (45.2)	8.710	0.003
	No	175 (58.3)	23 (37.7)	198 (54.8)		
Presence of termite mounds	Yes	87 (29.0)	40 (65.6)	127 (35.2)	29.736	< 0.0001*
	No	213 (71.0)	21 (34.4)	234 (64.8)		

*P < 0.01

### Seroprevalence of VL and association with socio-demographic factors

Seroprevalence of VL varied relative to type of screening test wherein a higher prevalence was observed using rK39 (15.8%) than the ELISA (3.0%). Compared to the ELISA (confirmatory) test, rK39 exhibited a high frequency (80.7%) of seropositive results and the two tests showed fair agreement (κ = 0.287, *P* < 0.001) (Table 2).

**Table 2:** Comparative summary of overall VL seroprevalence based on rK39 and ELISA tests

Serological test		ELISA			*κ*	*P*-value
		Negative	Positive	Total		
rK39-ICT	Negative	304 (100.0)	0 (0)	304 (84.2)	0.287	< 0.0001
	Positive	46 (80.7)	11 (19.3)	57 (15.8)		
	Total	350 (97.0)	11 (3.0)	361 (100)		

On both serological tests, prevalence of VL was higher (*P* < 0.05) in Adadle than Gode district. The prevalence of VL based on rK39 test also increased (*P* < 0.050) with outdoor sleeping habit, raising livestock, as well as with the presence of *Acacia* trees and termite mounds in the study areas. The latter three and house type showed similar relative effect on prevalence of VL based on ELISA (*P* > 0.050) (Table 3).

**Table 3:** Association of socio-demographic factors and study districts with seroprevalence rate of VL

Variable	Category	rk39-ICT			ELISA		
		No. positive (%)	*χ* ^2^	*P*-value	No. positive (%)	*χ* ^2^	*P*-value
Study area	Gode	38 (12.7)	13.021	< 0.0001*	6 (2.0)	6.589	0.010
	Adadle	19 (31.1)			5 (8.2)		
Sex	Female	35 (17.2)	0.660	0.417	7 (3.4)	0.234	0.628
	Male	22 (14.0)			4 (2.5)		
Age group	0–14	28 (20.4)	4.097	0.251	6 (4.4)	1.683	0.641
	15–29	15 (14.3)			3 (2.9)		
	30–44	8 (10.5)			1 (1.3)		
	> 44	6 (14.0)			1 (2.3)		
Occupation	Farmer	19 (16.1)	8.353	0.079	4 (3.4)	2.432	0.657
	House worker/wife	4 (6.5)			1 (1.6)		
	Student/cattle keeper	30 (21.3)			6 (4.3)		
	Employed	1 (7.7)			0 (0)		
	Private	3 (11.1)			0 (0)		
House type (wall)	Mud/wood	39 (15.1)	0.526	0.769	10 (3.9)	2.253	0.324
	Mud/stone	12 (18.8)			1 (1.6)		
	Wood	6 (15.8)			0 (0)		
Sleeping outdoors	Yes	40 (20.8)	7.847	0.005	6 (3.1)	0.008	0.927
	No	17 (10.1)			5 (3.0)		
Domestic animal	Yes	54 (19.4)	11.743	0.001*	10 (3.6)	1.200	0.273
	No	3 (3.7)			1 (1.2)		
*Acacia* tree	Yes	47 (28.8)	38.043	< 0.0001*	7 (4.3)	1.565	0.211
	No	10 (5.1)			4 (2.0)		
Termite mounds	Yes	44 (34.6)	52.393	< 0.0001*	5 (3.9)	0.525	0.469
	No	13 (5.6)			6 (2.6)		

*P < 0.01

Females and children (0–14 years of age) who sleep outdoors (*χ*^2^ = 4.182, *P* = 0.041 *vs χ*^2^ = 11.035, *P* = 0.012), and those who engaged in cattle keeping (*χ*^2^ = 6.281, *P* = 0.179 *vs χ*^2^ = 26.392, *P* = 0.009) showed higher VL seropositive reaction by rK39 test than their respective counterparts (see Additional file 1: Figure S1 and Additional file 2: Figure S2).

### Factors associated with VL seroprevalence

Logistic-regression analysis of factors exhibiting significant independent effect on seroprevalence of VL based on the rK39 test revealed a significant effect for the presence of termite mounds and *Acacia* trees, outdoor sleeping habit, study district and owning domestic animals (Table 4).

**Table 4:** Logistic regression model output for factors associated with VL in the study districts

Variable	Category	*n*	No. positive (%)	Exp (B)	95% CI	*P*-value
Study area	Gode	300	38 (12.7)	5.404	2.900–10.070	< 0.0001*
	Adadle	61	19 (31.1)			
Outdoor sleeping	Yes	192	40 (20.8)	5.313	2.283–12.364	< 0.0001*
	No	169	17 (10.1)			
Domestic animals	Yes	279	54 (19.4)	0.199	0.097–0.410	< 0.0001*
	No	82	3 (3.7)			
*Acacia* tree	Yes	163	47 (28.8)	2.372	1.1190–4.728	0.014
	No	198	10 (5.1)			
Termite mounds	Yes	127	44 (34.6)	12.578	5.911–26.763	< 0.0001*
	No	234	13 (5.6)			

*P** < **0.01

### Composition and distribution of phlebotomine sand flies

**Fig. 2:**
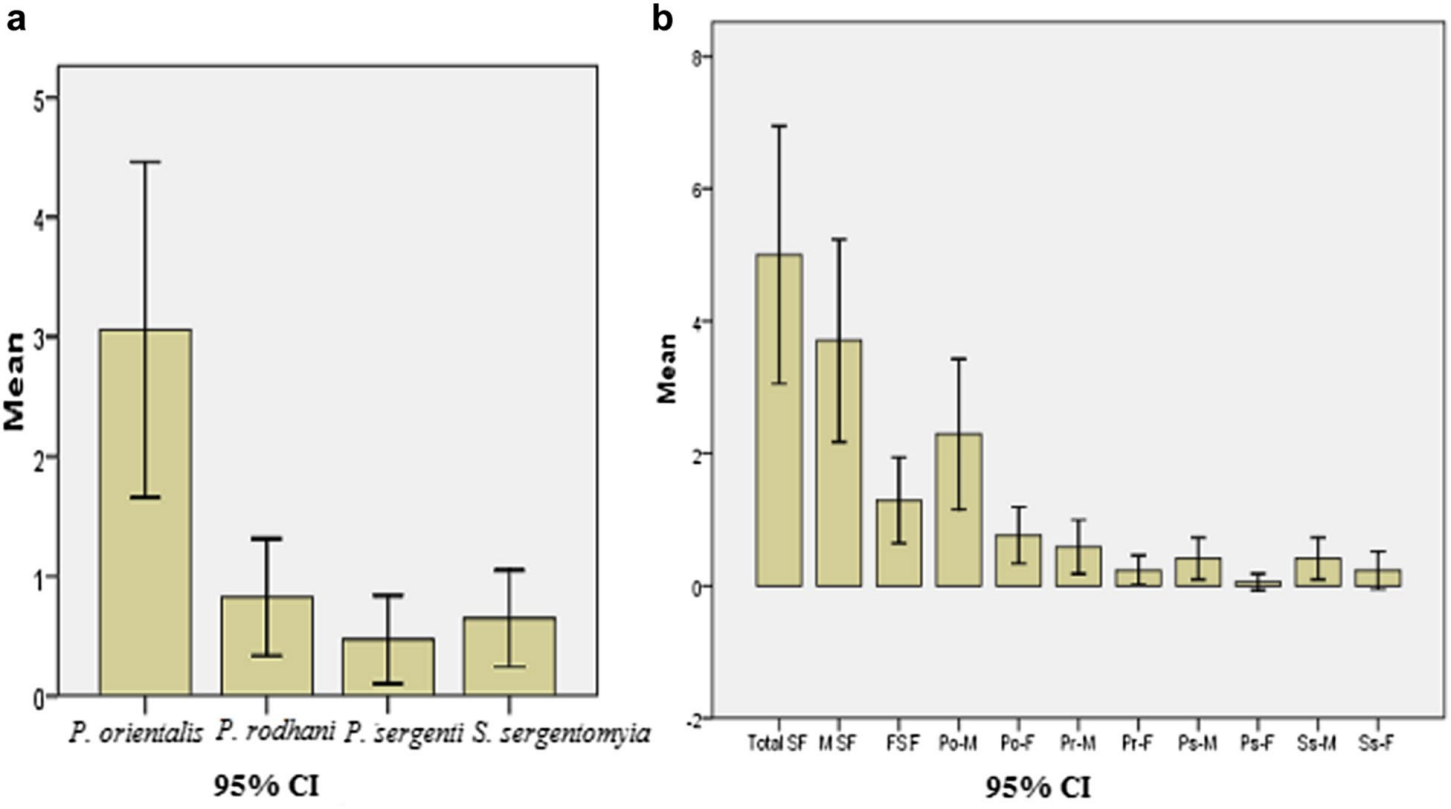
Average frequency of sand fly by species (**a**) and sex (**b**). *Abbreviations*: SF, sand fly; MSF, male sand fly; FSF, female sand fly; Po-M, male *P. orientalis*; Po-F, female *P. orientalis*; Pr-M, male *P.rodhaini*; Pr-F, female *P.rodhaini*; Ps-M, male *P.sergenti*; Ps-F, female *P.sergenti*; Ss-M, male *S.sergentomyia*; Ss-F, female *S. sergentomyia*

A total of 74 *Phlebotomus* spp. and 11 *Sergentomyia* spp. sand flies were identified including *P.* (*Larroussius*) *orientalis* (52, 61.2%), *P.* (*Anaphlebotomus*) *rodhaini* (14, 16.2%), *P.* (*Paraphlebotomus*) *sergenti* (8, 9.4%) and *S.sergentomyia* (unclassified) (11, 12.9%). The average frequency of *P. orientalis* (3.06 ± 0.66) was higher (*P* = 0.050) than that of the other sand fly species (Fig. 2a). Similarly, the average total frequency of male sand flies (3.71 ± 0.721) was higher (*P* = 0.001) than that of females (1.29 ± 0.326) (Fig. 2b).

The average frequency of total and specific sand fly species showed a slight (*P* > 0.050) variation relative to study district and fly trapping method. The average frequency of female sand flies was higher (*P* = 0.038) in Adadle district (1.89 ± 0.423) than in Gode district (0.62 ± 0.324). This trend was more pronounced in *P. orientalis* (1.11 ± 0.309 *vs* 0.38 ± 0.183) (see Additional file 3: Figure S3). Adadle district also accounted for a higher proportion of total and of specific sand fly species than Gode district (Table 5).

**Table 5:** Species composition and distribution of phlebotomine sand flies

Factor	Group	*N* (%)	*P. orientalis* *n* (%)	*P. rodhaini* *n* (%)	*P. sergenti* *n* (%)	*S. sergentomyia* *n* (%)
District	Gode	26 (30.6)	18 (34.6)	4 (28.6)	1 (12.5)	3 (27.3)
	Adadle	59 (69.4)	34 (65.4)	10 (71.4)	7 (87.5)	8 (72.7)
Site	In doors	3 (3.5)	0 (0)	0 (0)	1 (12.5)	2 (18.2)
	Mound	32 (37.7)	24 (46.1)	4 (28.6)	3 (37.5)	1 (9.1)
	Tree	16 (18.8)	7 (13.5)	2 (14.3)	2 (25.0)	5 (45.4)
	Cattle shed	23 (27.1)	13 (25.0)	6 (42.8)	2 (25.0)	2 (18.2)
	Artificial pond	11 (12.9)	8 (15.4)	2 (14.3)	0 (0)	1 (9.1)
Trap	CDC light	40 (47.1)	24 (46.1)	7 (50.0)	4 (50.0)	5 (45.5)
	Sticky	34 (40.0)	20 (38.5)	5 (35.7)	4 (50.0)	5 (45.5)
	Aspiration	11 (12.9)	8 (15.4)	2 (14.3)	0 (0)	1 (9.1)

On the other hand, a higher proportion of sand flies were captured in outdoor traps. Moreover, the average frequency of total sand flies was higher (*P* = 0.012) in termite mounds (8 ± 1.5) and cattle sheds (5.75 ± 1.9) than other sampling sites. Likewise, the average *P. orientalis* frequency was higher (*P* < 0.001) near termite mounds (6 ± 0.913) compared to other sites. The average frequency of male *Sergentomyia* sand flies was notably higher (*P* = 0.001) near *Acacia* trees (Mean ± SE = 1.25 ± 0.250) (see Additional file 3: Figure S3).

## Discussion

This study is the first detailed epidemiological investigation on seroprevalence, associated factors and phlebotomine sand fly vectors of VL in the two study districts and Ethiopian Somali Region in general. The overall prevalence of VL in the study area was estimated at 15.8% based on the rK39 test and 3.0% using the ELISA test. The latter seroprevalence estimate may be lower due to lapses in optimum specimen collection and processing protocols attributed to distance and transport challenges between study districts and laboratories located at the Black Line Hospital (Addis Ababa University Medical School) and Konso station in southern Ethiopia. Moreover, others have reported that the ELISA method underestimates VL positivity in cases of immune-compromising conditions such as malnutrition, and co-infections with HIV, malaria and other parasites [26–29]. On the other hand, the current prevalence of VL in both rK39 and ELISA tests was higher than previous estimates of 1.02–13.0% [30–35] and 1.8% [3] reported from other parts of Ethiopia. Migratory and outdoor lifestyles, and common chronic food-insecurity related malnutrition problems in pastoralists have been incriminated as major factors for contracting VL in well-established local disease foci [7, 8, 10, 36].

A marked difference in total VL prevalence based on both rK39 and ELISA serological tests were observed between Adadle (31.1 *vs* 8.2%) and Gode (12.7 *vs* 2.0%) districts. This difference could be attributed to higher livestock keeping trends, *Acacia*/vegetation cover and the presence of termite mounds in Adadle district. These factors, coupled with outdoor sleeping habits (in both districts), were associated with a higher (*P* < 0.050) seroprevalence of VL based on the rK39 test, whereas similar trends were limited for the ELISA test, owing largely to the low frequency of positive reactions in different risk groups. Termite mounds and *Acacia* vegetation provide favorable breeding/resting sites for *Phlebotomus* species [10, 31, 37]. Cattle sheds tend to be attractive to vector sand flies whereas stock herding increases risk of exposure to vectors that are associated with range plants. In agreement with current observations, others have indicated that a higher risk of VL infection is associated with the presence of termite mounds, outdoor sleeping, *Acacia* trees and livestock keeping [12, 38–43].

In this study, the seroprevalence of VL based on the rK39 test was relatively more elevated (*P* > 0.05) in females and children under the age of 15 years than in males and in other age groups. In contrast, others have previously reported a higher risk of VL in males than in females under the age of 15 [3, 6, 9, 30, 31]. Earlier studies in southeastern semi-arid areas of the Ethiopian Somali Region [8] and elsewhere [37, 44, 45] have shown a higher risk of VL in children under 15 years compared to adults. Gender- and age-related predisposition to VL infection could vary from society to society depending on prevailing household labor-division trends. In the present study area, it was observed that children under 15 years (both male and female) are responsible for herding livestock in vector-infested range lands, whereas women carry the heavier burden of household chores (fetching water, gathering wood fire, milking and food preparation), some of which may contribute to increased exposure to fly vectors. Furthermore, the occurrence of acute malnutrition among children of pastoralist communities that commonly has been reported is thought to be a risk factor for the disease. In the context of the whole of Ethiopia, children who previously had malnutrition are reported to be more susceptible to the disease [46]. According to information obtained from Gode hospital, VL positive children from the two districts were treated in the hospital. A comparison of previous hospital records data may reveal an increase in positive cases among children, providing further evidence of disease transmission within the study sites.

Despite the fact that a small number of phlebotomine sand fly samples were collected, the preliminary entomological survey identified four potential sand fly vectors, namely *P. orientalis* (61.2%), *P.rodhaini* (16.2%), *S.Sergentomyia* (12.9%) and *P.sergenti* (9.4%). *P. orientalis* is a proven vector of *L.donovani* in many endemic areas of Ethiopia [11, 47–50]. *Phlebotomussergenti* is recognized as a potential vector of cutaneous leishmaniasis due to *Leishmania tropica* in Ethiopia [51–53]. Meanwhile, *P.rodhani* is suspected as a VL vector in Sudan and implicated in maintaining the zoonotic cycle between reservoir animals and humans [54]. *P. orientalis* and *P.rodhani* appear to be potential primary and secondary vectors of VL in the study area*.* The frequency of total and female sand flies was higher in Adadle district. This could be attributed to the greater availability of suitable resting and breeding habitats such as termite mounds, *Acacia* trees, and livestock rearing in the district. In-line with this, *Phlebotomus* species were captured more frequently from termite mounds followed by cattle sheds. The study findings agree with those reported by many others [11, 20, 55, 56]. The ventilation shafts of termite mounds provide optimum resting and breeding sites for these flies. The presence of numerous female *P. orientalis* (some of which were blood engorged) and *P.rodhaini* near animal sheds indicate their feeding preference towards domestic animals. This trend is consistent with other studies [11, 42, 56]. However, considering the relatively scant number of collected sand fly samples, which might be associated with seasonality and duration of the study period, further entomological and laboratory investigations are required. These are needed to establish with certainty the role of these two potential vectors in view of their importance on the epidemiology of VL in the study area as well as in the Somali Region in general.

## Conclusions

The present study demonstrates that the seroprevalence rate of VL is alarmingly higher in Adadle than in Gode district. Females and children under the age of 15 years showed higher VL seroreactivity compared to their respective counter groups. *P. orientalis* appears to be the potential primary vector of VL. Higher livestock keeping trends, *Acacia* vegetation cover, outdoor sleeping habits and the presence of termite mounds were associated with increased VL seroprevalence. Generally, the presence of numerous seropositive children and the abundance of *P. orientalis* and favorability of the ecology to sand flies coupled with earlier hospital-based data of detected active cases on children, strongly enlighten possible transmission of VL in the study areas. Many questions remain to be elucidated concerning transmission of VL in the study areas. First, molecular characterization of the parasite in humans, sand fly vectors and reservoir hosts, then determination of the feeding preference of sand fly vectors and its ability to harbor, develop and transmit *Leishmania* parasite to a susceptible host is required to confirm the transmission of the disease. Concomitantly, understanding the transmission pattern of VL, including seasonal abundance of sand flies and the seroprevalence rate of VL in the study districts and the nearby localities will be of paramount importance for more focused interventions and to control the spread of the disease.

## Additional files

**Additional file 1: Figure S1.** Association of VL with sex (**a**) and age (**b**) of participants relative to outdoor sleeping habit.

**Additional file 2: Table S1.** Association of VL with sex (**a**) and age (**b**) of participants relative to occupation.

**Additional file 3: Table S2.** Relative frequencies of sand flies relative to district (**a**) and site (**b**).

## Abbreviations

VL: visceral leishmaniasis
rK39-ICT: rK39-immunochromatographic test
ELISA: enzyme-linked immunosorbent assay
DAT: direct agglutination test
CDC: Centers for Disease Control and Prevention
ST: sticky trap
O.D.: optical density
CI: confidence interval
