# Supplementary figures and images for: Epidemiology of visceral leishmaniasis in Shebelle Zone of Somali Region, eastern Ethiopia
Source: Parasit Vectors. 2019 May 6;12:209. doi: 10.1186/s13071-019-3452-5 (PMC6503444; doi:10.1186/s13071-019-3452-5)

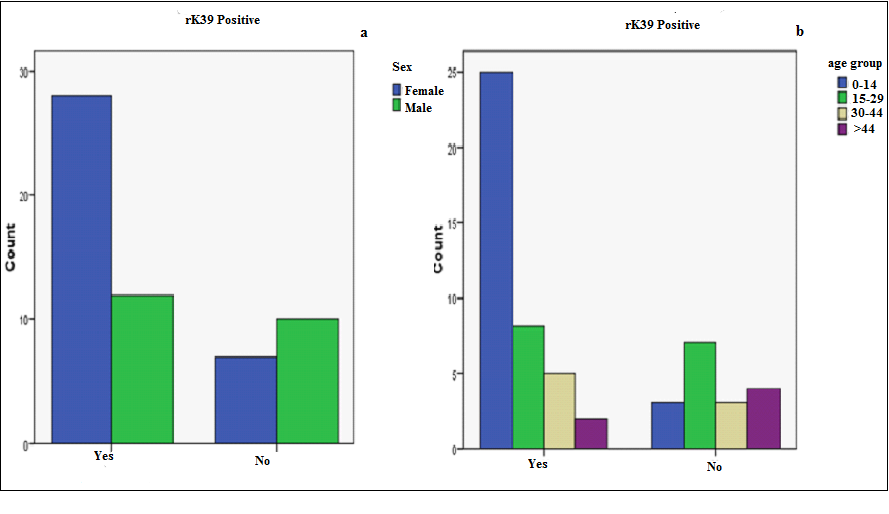

Supplement: Supplementary file 1 — Additional file 1: Figure S1. Association of VL with sex (a) and age (b) of participants relative to outdoor sleeping habit. [file 13071_2019_3452_MOESM1_ESM.tif]

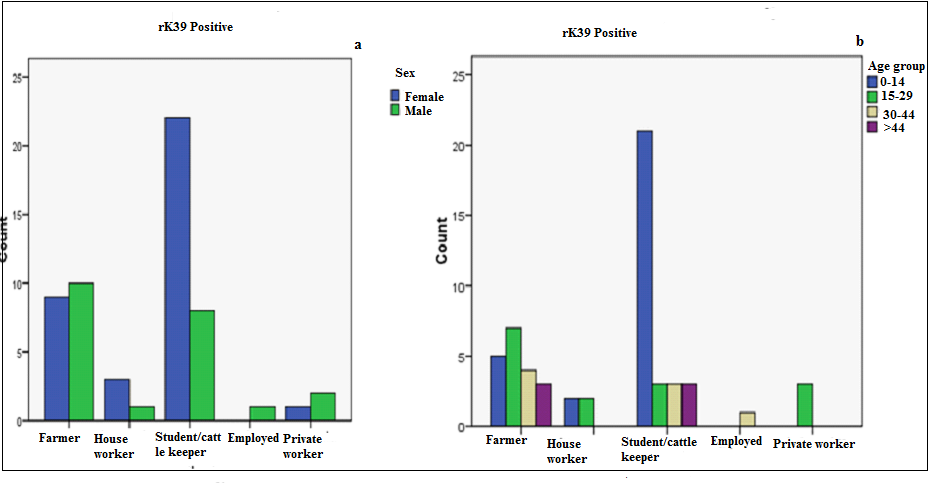

Supplement: Supplementary file 2 — Additional file 2: Table S1. Association of VL with sex (a) and age (b) of participants relative to occupation. [file 13071_2019_3452_MOESM2_ESM.tif]

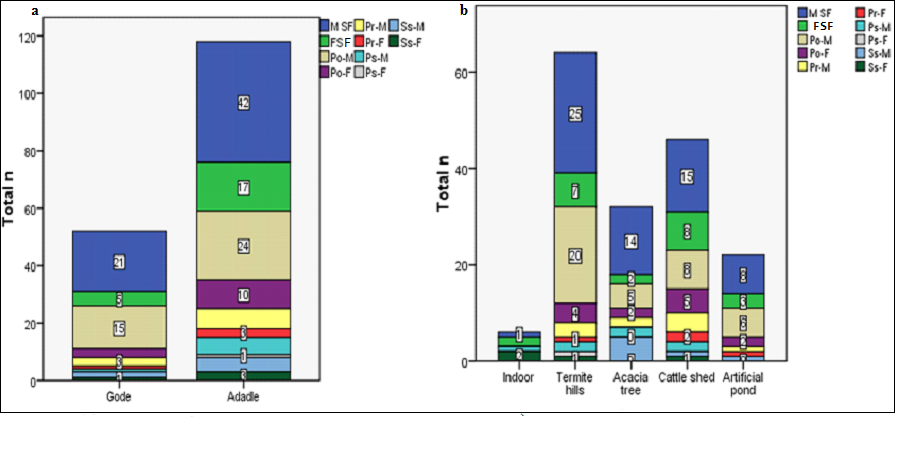

Supplement: Supplementary file 3 — Additional file 3: Table S2. Relative frequencies of sand flies relative to district (a) and site (b). [file 13071_2019_3452_MOESM3_ESM.tif]
